# A Hydrogen Gas Sensor Based on TiO_2_ Nanoparticles on Alumina Substrate

**DOI:** 10.3390/s18082483

**Published:** 2018-08-01

**Authors:** Siti Amaniah Mohd Chachuli, Mohd Nizar Hamidon, Md. Shuhazlly Mamat, Mehmet Ertugrul, Nor Hapishah Abdullah

**Affiliations:** 1 Institute of Advanced Technology, University Putra Malaysia, Serdang 43400, Selangor, Malaysia; mnh@upm.edu.my (M.N.H.); hapishah@upm.edu.my (N.H.A.); 2 Faculty of Electronic & Computer Engineering, Universiti Teknikal Malaysia Melaka, Hang Tuah Jaya, Durian Tunggal 76100, Melaka, Malaysia; 3 Faculty of Sciences, University Putra Malaysia, Serdang 43400, Selangor, Malaysia; shuhazlly@upm.edu.my; 4 Engineering Faculty, Ataturk University, 25240 Erzurum, Turkey; ertugrul@atauni.edu.tr

**Keywords:** gas sensor, TiO_2_-B_2_O_3_, hydrogen, nanoparticles, p-type TiO_2_

## Abstract

High demand of semiconductor gas sensor works at low operating temperature to as low as 100 °C has led to the fabrication of gas sensor based on TiO_2_ nanoparticles. A sensing film of gas sensor was prepared by mixing the sensing material, TiO_2_ (P25) and glass powder, and B_2_O_3_ with organic binder. The sensing film was annealed at temperature of 500 °C in 30 min. The morphological and structural properties of the sensing film were characterized by field emission scanning electron microscopy (FESEM), energy-dispersive X-ray spectroscopy (EDX) and X-ray diffraction (XRD). The gas sensor was exposed to hydrogen with concentration of 100–1000 ppm and was tested at different operating temperatures which are 100 °C, 200 °C, and 300 °C to find the optimum operating temperature for producing the highest sensitivity. The gas sensor exhibited p-type conductivity based on decreased current when exposed to hydrogen. The gas sensor showed capability in sensing low concentration of hydrogen to as low as 100 ppm at 100 °C.

## 1. Introduction

Detection of hydrogen in fuel cell, combustion engines and monitoring faults in transformer have gained incredible interest from many researchers especially from gas sensing area. Hubert et al. reported that 1400 publications have been published in gas sensing from 1975 until 2010 [1]. Hydrogen which is known as a colorless, odorless, tasteless, and flammable gas, cannot be detected by human senses [2], thus its presence should be detected and analyzed. With a mixture of oxygen, leakage of hydrogen can cause explosions and degradation of many types of steels [3]. Hydrogen can also become flammable and explosive if the concentration is higher than 4% in air [4].

Different sensing technologies have been employed to detect hydrogen, such as catalyst, thermal conductivity, electrochemical, resistance based, work function based, mechanical, and optical [2]. Among them, electrochemical and resistance-based technologies are the most preferred due to their ability to detect low hydrogen concentration and acceptable selectivity [2]. It has been reported that effective sensing materials to sense hydrogen are based on palladium (Pd) [5,6,7,8,9,10,11,12,13,14,15] and metal-oxide semiconductors (MOX) such as SnO_2_ [16,17,18,19], ZnO [20,21,22,23], TiO_2_ [24,25,26,27,28,29,30], WO_3_ [31], and NiO [32] because of their capability to detect hydrogen with low concentration and ability to work at room temperature. Palladium is high sensitive to hydrogen; however it also has drawbacks such as hysteresis behavior in electrical resistance because of adsorption of hydrogen in the structure of Pd [5]. Recently, a hydrogen gas sensor based on carbon-based materials such as carbon nanotubes [33,34,35], graphene [36,37,38,39], and reduced graphene oxide (RGO) [40,41] has also attracted high attention because it is highly sensitive to the changes in the chemical environments [42,43], and offers high performance, label free chemical sensing [44].

TiO_2_ has been chosen in this work because it is known as a chemically stable, nontoxic, biocompatible, inexpensive, wide band gap semiconducting material [45]. Due to being inexpensive, hydrogen gas sensors based on TiO_2_ also can become affordable and safe hydrogen gas sensors [46]. Among the metal-oxide semiconductors, the TiO_2_ gas sensor has been reported to be able to work under low operating temperatures, up to as low as room temperature [28,47,48], with fast response [29]. These criteria have made TiO_2_ a practical material for gas sensing applications.

In this paper, a TiO_2_ gas sensor was fabricated on the alumina substrate using screen-printed method and was tested to different concentration of hydrogen from 100–1000 ppm at three different operating temperatures: 100 °C, 200 °C, and 300 °C.

## 2. Materials and Methods

### 2.1. Preparation and Fabrication of Gas Sensor

**Figure 1:**
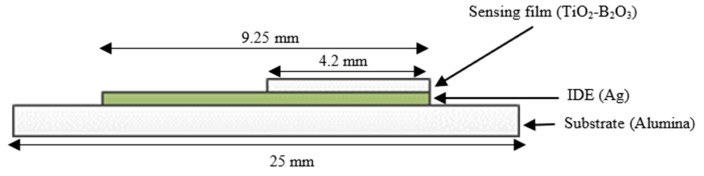
Front view of the TiO_2_-B_2_O_3_ gas sensor.

Gas sensor used in this work consist of two layers, which are an interdigitated electrode (IDE) and sensing film, as shown in Figure 1. The IDE used in this work was a silver-conductive paste (DGP80 TESM8020) provided by Sigma-Aldrich (Steinheim am Albuch, Germany) and the sensing material used in this work was TiO_2_ (Aeroxide^®^ P25) provided by Sigma-Aldrich (Steinheim am Albuch, Germany). IDE and the sensing film were deposited on the alumina substrate using a screen printing method. Initially, IDE was deposited as first layer on the alumina substrate, followed by annealing in the furnace at temperature of 120 °C for 30 min. Air was used as a carrier gas in the furnace.

**Figure 2:**
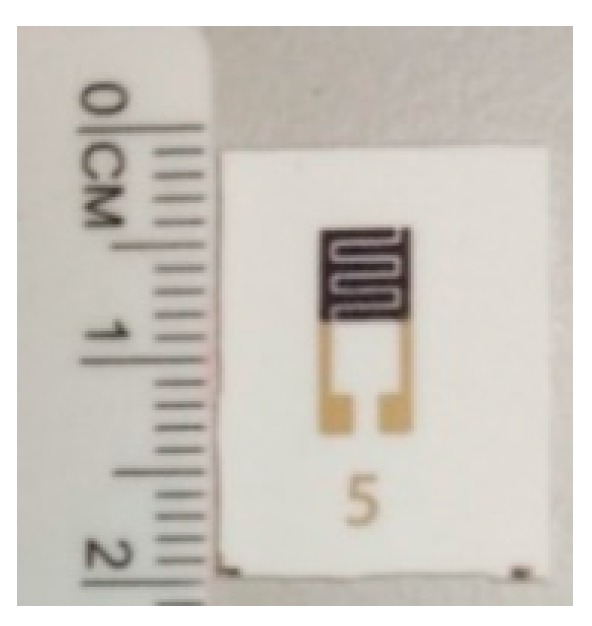
Fabricated TiO_2_-B_2_O_3_ gas sensor on alumina substrate using a screen-printing method.

In order to deposit TiO_2_ on the alumina substrate, TiO_2_ powder was prepared as a paste. Firstly, 90 wt % of TiO_2_ powder was mixed with 10 wt % of glass powder, boron oxide (B_2_O_3_), using m-xylene as a medium in an ultrasonic bath for 90 min. Then, it was dried in an oven and was ground in a mortar. The purpose of glass powder is to hold the nanoparticles of TiO_2_ on the substrate and to ensure good adhesion between TiO_2_ and the alumina substrate. B_2_O_3_ was chosen as the glass powder in this work because it has a low melting point of 450 °C. This method have been presented in [49,50]. Organic binder was prepared by mixing m-xylene, linseed oil, and α-terpineol. The paste was prepared by mixing the TiO_2_-B_2_O_3_ with organic binder until homogeneous paste was obtained. Then, TiO_2_-B_2_O_3_ paste was deposited on the top of IDE and it was annealed in the furnace at temperature of 500 °C for 30 min. The fabricated gas sensor is shown in Figure 2. The size of the sensing film was 4.2 × 4.2 mm, while the size of the IDE was 9.25 × 4.2 mm. The IDE was fully covered by TiO_2_-B_2_O_3_ paste in order to increase the sensitivity of the gas sensor. Black color on the sensing film of gas sensor might be caused by diffusion of silver (IDE) into the TiO_2_ (Figure 2). It was reported that the silver diffused into TiO_2_ at a temperature of 400 °C [51].

### 2.2. Characterization Method of TiO_2_-B_2_O_3_

Characterizations of TiO_2_-B_2_O_3_ were made using a thermogravimetric analyzer (TGA), field emission scanning electron microscopy (FESEM), energy-dispersive X-ray spectroscopy (EDX), and X-ray diffraction (XRD). Thermal analysis of TiO_2_-B_2_O_3_ paste was tested using TGA (Brand: Mettler Toledo (Greifensee, Switzerland, Model: TGA/DSC 1 HT)) with heating rate 10 °C/min and air as the carrier for the temperature range: 25–1000 °C. The surface morphology of the thick films was analyzed using FESEM (Model: Nova Nanosem 230 (Thermo Fisher Scientific, Oregon City, OR, USA), and element composition was examined by EDX inside the FESEM. XRD (Brand: Philips (Almelo, The Netherlands), Model: PW 3040/60 MPD X’pert High Pro Panalytical) studies were carried out for powder and thick film over a 2θ range from 20° to 80°. The scanning time for TiO_2_ (P25) and TiO_2_-B_2_O_3_ powder were 5 min, and one hour for TiO_2_-B_2_O_3_ thick films.

### 2.3. Gas Response Measurement

**Figure 3:**
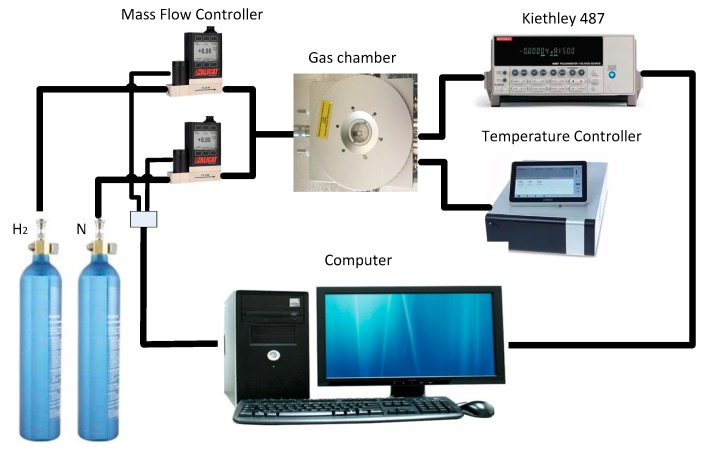
Experimental setup of gas sensing measurement.

Gas sensing measurements were performed in gas chamber with different ppm levels of hydrogen from 100–1000 ppm. As a carrier gas, 500 sccm nitrogen was used. Experimental setup of gas chamber is shown in Figure 3. Gas chamber was obtained from Linkam Scientific (Tadworth, UK, Model: HFS600). The gas chamber was connected to the mass flow controller, temperature controller, and Kiethley 487 Picoammeter/Voltage source. Three different operating temperatures were tested on gas sensor which are 100 °C, 200 °C, and 300 °C to find the optimum operating temperature that can produce highest sensitivity to hydrogen. For measurement, 10 V voltage source was applied to the IDE of gas sensor and current was observed as the response of gas sensor.

## 3. Results and Discussion

### 3.1. Characterization of TiO_2_-B_2_O_3_ Using TGA, FESEM, EDX and XRD

**Figure 4:**
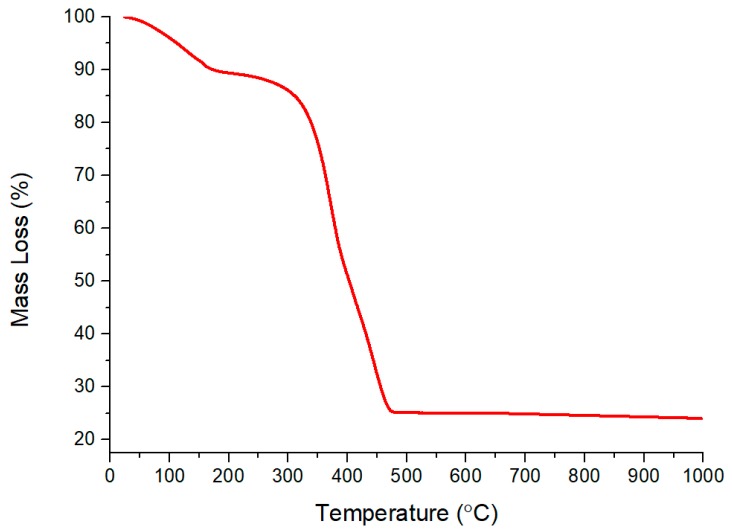
Thermal Behavior of TiO_2_-B_2_O_3_ paste using thermogravimetric analysis (TGA).

TGA analysis was performed to determine the thermal behavior of the TiO_2_-B_2_O_3_ paste and to find an optimum calcination temperature. Figure 4 shows total mass loss of TiO_2_-B_2_O_3_ paste over a temperature range of 25 to 1000 °C. At 400 °C, mass loss was measured approximately 49.61%, which indicated that the organic binder was not fully evaporated at this temperature. It became decreased again when temperature reached at 500 °C which approximately 25.12%. Composition ratio of TiO_2_-B_2_O_3_ powder and organic binder used in this work was 30:70. It can be seen that the organic binder was fully evaporated at temperature of 500 °C. Therefore, this temperature has been chosen as the annealing temperature for the sensing film.

**Figure 5:**
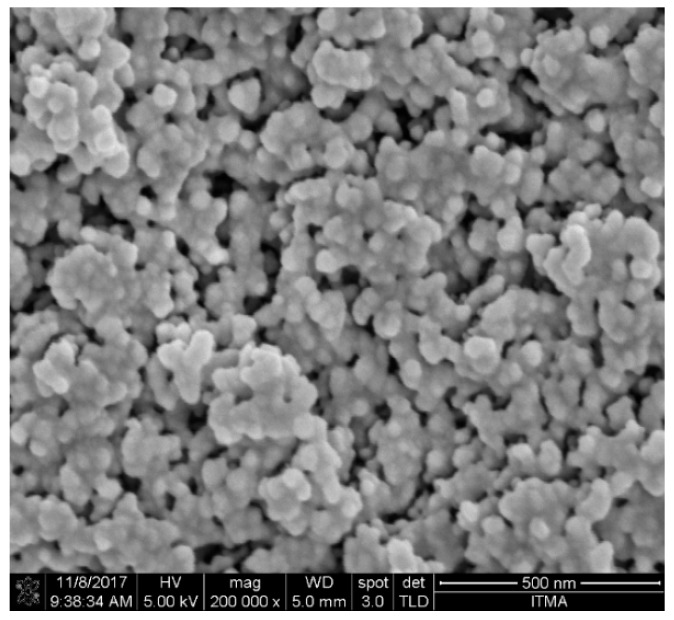
Field emission scanning electron microscopy (FESEM) image of TiO_2_-B_2_O_3_ on the alumina substrate at T = 500 °C.

**Figure 6:**
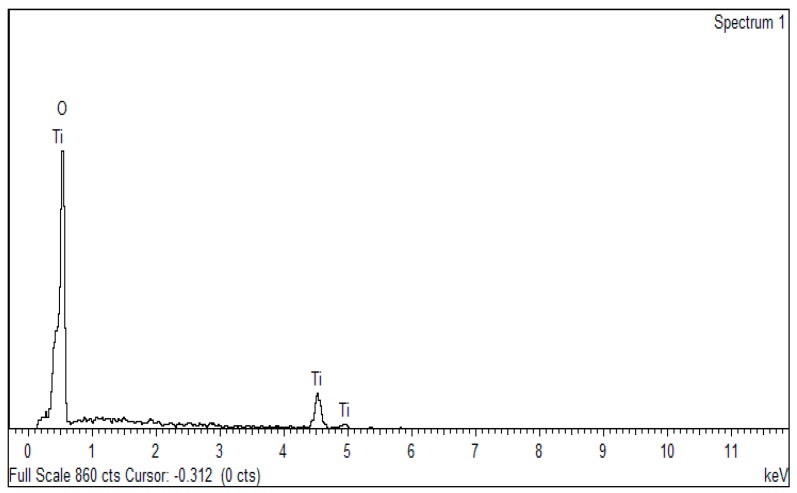
Energy-dispersive X-ray (EDX) results of TiO_2_-B_2_O_3_ on the alumina substrate at T = 500 °C.

The morphology of the TiO_2_-B_2_O_3_ nanoparticles (NP) structure at a temperature of 500 °C is shown in Figure 5. It can be seen that the nanoparticles of TiO_2_ was clearly seen at 200k magnification. Field emission scanning electron microscopy (FESEM) images shown the uniformity of nanostructures due to the homogeneity of prepared paste. Average diameter of nanoparticles was observed to be in 40–70 nm. The EDX result showed that peak of Ti was detected at temperature of 500 °C as shown in Figure 6. These results confirmed that TiO_2_ was crystalline at this temperature. Thus, it has been chosen as the sensing film of the gas sensor, and will be tested with hydrogen exposure in gas chamber.

**Figure 7:**
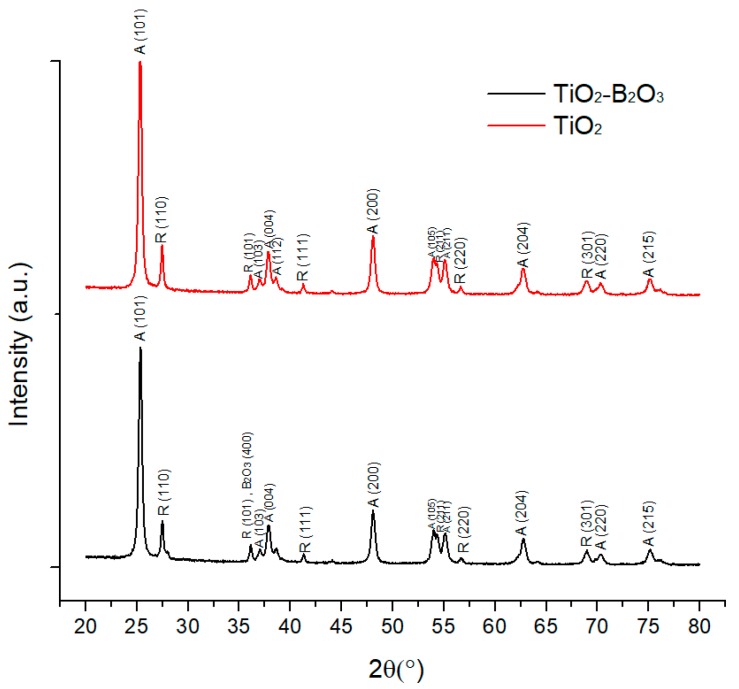
X-ray diffraction pattern of TiO_2_ (P25) and TiO_2_-B_2_O_3_.

**Figure 8:**
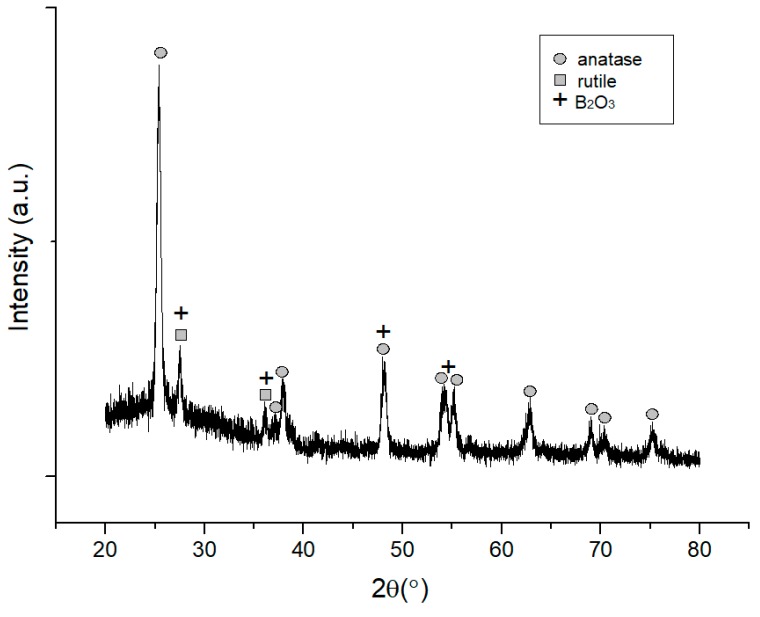
X-ray diffraction pattern of TiO_2_-B_2_O_3_ thick film at T = 500 °C.

Figure 7 shows the XRD pattern of TiO_2_ (P25) and TiO_2_-B_2_O_3_ without heat treatment. XRD analyses were examined using X’Pert HighScore software. From XRD spectra, it can be seen that both figures (TiO_2_ and TiO_2_-B_2_O_3_) consisted of rutile and anatase phases. It can be observed that pure titanium had a peak at 2θ = 25.35° (Figure 7 and Figure 8). According to literature, this peak was attributed to the anatase (101) TiO_2_ phase. Whereas, a high peak of rutile phase (110) was located at 2θ = 27.49° (Figure 7 and Figure 8). It was reported that crystallinity of TiO_2_ was decreased with the addition of boron content [52]. This work also showed that the intensity of TiO_2_ was decreased when added with B_2_O_3_. It can also be seen that the peak of anatase (101) in TiO_2_-B_2_O_3_ was lower than peak of anatase (101) in TiO_2_ at 2θ = 25.35°. Meanwhile, a small peak of B_2_O_3_ was observed at 2θ = 36.04° in TiO_2_-B_2_O_3_. This phase also contributed to the rutile phase (101).

Figure 8 shows XRD pattern of TiO_2_-B_2_O_3_ thick film at T = 500 °C. It can be seen that, XRD pattern of anatase and rutile phases in TiO_2_-B_2_O_3_ thick film was similar as XRD pattern in Figure 7. Whereas, B_2_O_3_ peaks were also detected in thick film at 2θ = 27.76°, 36.04°, 48.37° and 54.58°. It was also observed that peaks of B_2_O_3_ were detected at similar location of anatase (2θ = 48.37°) and rutile phases (2θ = 27.76°, 36.04° and 54.58°). This analysis also indicated that the XRD pattern of TiO_2_ was not affected by the presence of B_2_O_3_.

### 3.2. Electrical Characteristics of TiO_2_-B_2_O_3_ Gas Sensor

**Figure 9:**
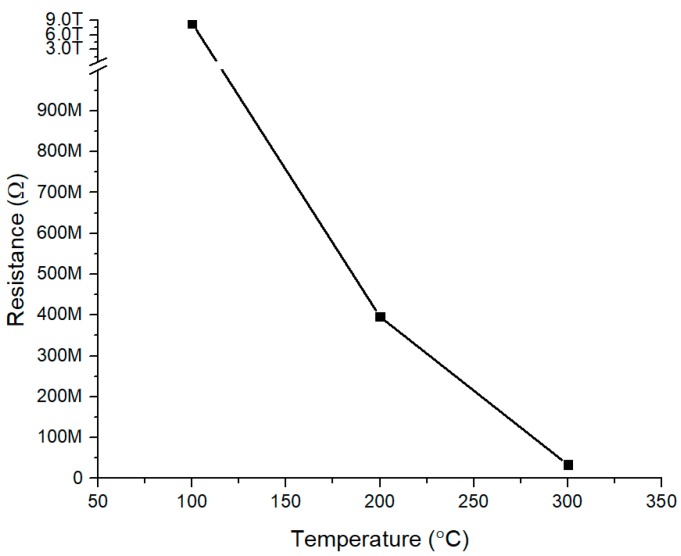
Electrical Characteristics of TiO_2_-B_2_O_3_ at different operating temperature.

Electrical characteristics of the TiO_2_-B_2_O_3_ gas sensor that annealed at 500 °C were studied. Figure 9 shows the resistance of TiO_2_-B_2_O_3_ gas sensor at the operating temperatures: 100 °C, 200 °C, and 300 °C. The graph showed that resistance was approximately 8.36 TΩ at 100 °C. This caused the range of current to be below than 1 pA. The resistance was dropped sharply at a temperature of 200 °C and 300 °C, where the values were approximately 39.59 GΩ and 33.74 MΩ respectively. This phenomenon can be caused by the conversion of silver (electrode) to metallic silver at operating temperatures of 200 °C and 300 °C, where it was decomposed into silver and oxygen [53]. This metallic silver has decreased the resistivity of the gas sensor and improved the conductivity of the gas sensor.

### 3.3. Performance of TiO_2_-B_2_O_3_ Gas Sensor at Different Operating Temperatures

**Figure 10:**
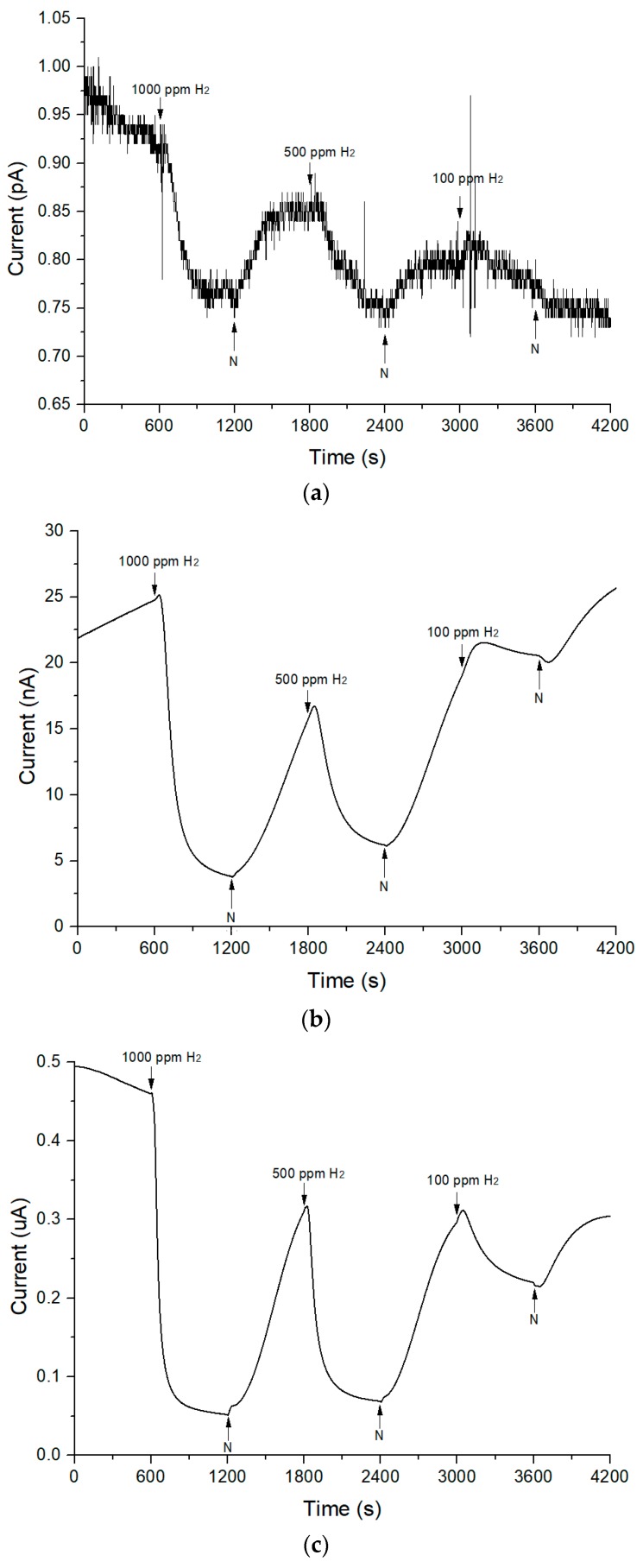
Response of TiO_2_-B_2_O_3_ gas sensor to hydrogen at different operating temperature (**a**) T = 100 °C (**b**) T = 200 °C (**c**) T = 300 °C.

Figure 10 shows the response of TiO_2_-B_2_O_3_ gas sensors at operating temperatures: 100 °C, 200 °C and 300 °C. It can be seen that in Figure 10a, the measurement was quite sensitive to noise when the experiment was carried out at 100 °C. It was observed that the response was not as smooth as the response in Figure 10b,c. This environment occurred because the measured current was very low, which is below than 1 pA. As the operating temperature increased, the observed current started to increase and showed high response to hydrogen. From experiments have been conducted, TiO_2_-B_2_O_3_ gas sensor able to sense low concentration of hydrogen as low as 100 ppm at 100 °C. However, it also has been observed that the TiO_2_-B_2_O_3_ gas sensor was unable to operate at room temperature. Response showed that the observed current was decreased when exposed to hydrogen and it was increased when exposed to the nitrogen. It also means that resistance of TiO_2_-B_2_O_3_ increased when exposed to the hydrogen and decreased when exposed to the nitrogen. This behavior indicated that TiO_2_-B_2_O_3_ gas sensor is a p-type gas sensor based on its response. P-type responses might be caused by diffusion of silver into TiO_2_. Sheini and Rohani [51] have compared the sensing mechanism of TiO_2_ to reducing gas before and after silver diffusion into TiO_2_ and found that sensing mechanism of gas sensor has been changed to p-type when silver diffused into TiO_2_. The sensitivity of p-type gas sensor can be calculated as follows [54]:

$$S=\frac{R_{H2}}{R_{N}}$$

where *R_H_*_2_ is resistance in hydrogen flow and *R_N_* is initial resistance in nitrogen flow.

**Figure 11:**
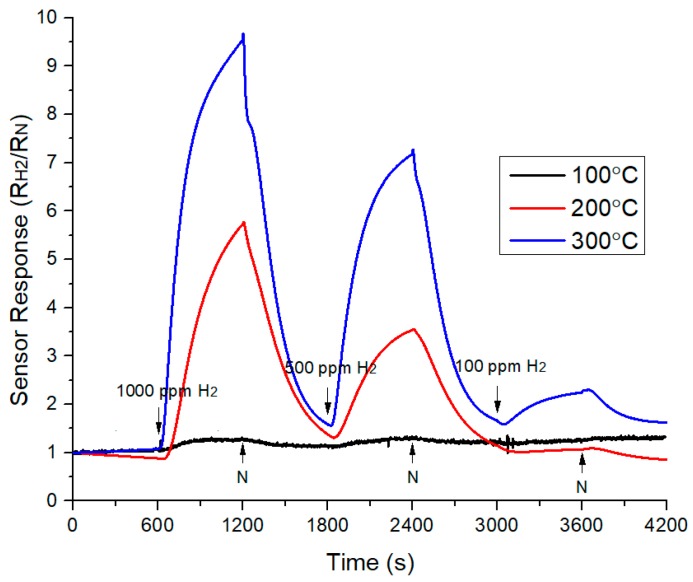
Sensor response of TiO_2_-B_2_O_3_ gas sensor at different operating temperature.

Comparison of sensor response at different operating temperature is shown in Figure 11. The gas sensor responded well to hydrogen. It also found that the responses values were unable to return to the original value, which is 1. This indicated that the responses were not fully recovered when nitrogen was flowed to the gas chamber. It can be seen that the value of sensor response was very low at an operating temperature of 100 °C compared to the operating temperature at 200 °C and 300 °C. The highest peak of sensor response was achieved at an operating temperature of 300 °C. The sensitivity was increased when the operating temperature was higher. The sensitivity of 100 ppm of H_2_ was the lowest at an operating temperature 200 °C due to the sensor response being the lowest at this temperature (Figure 10). Among three different temperatures, highest sensitivity was obtained at an operating temperature of 300 °C and the sensitivity values were 2.30, 7.28, and 9.68 at 100 ppm, 500 ppm, and 1000 ppm respectively. From observation, it can be concluded that resistance was decreased when temperature was increased. These indicated that flow of current will become higher as temperature increased, where more electrons can pass through the gas sensor and increase the conductivity. Overall, it can improve the sensitivity of the gas sensor.

**Figure 12:**
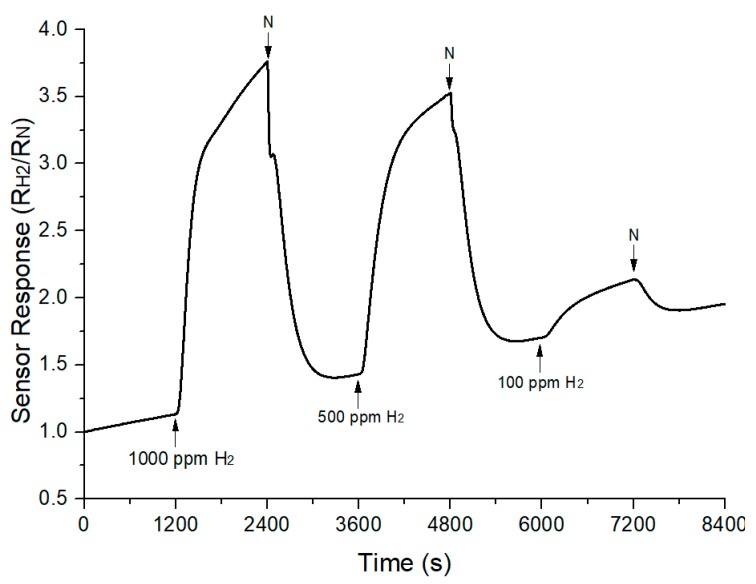
Sensor response of TiO_2_-B_2_O_3_ gas sensor to hydrogen at 300 °C.

In term of stability and repeatability properties of TiO_2_-B_2_O_3_ gas sensor, the same sample has been exposed to hydrogen at optimal operating temperature, which is at 300 °C. The cycle time of hydrogen and nitrogen was increased to 1200 s for this measurement. The sensor response of TiO_2_-B_2_O_3_ gas sensor is shown in Figure 12. It can be seen that the gas sensor was unable to recover well when exposed to hydrogen even though the cycle time has been increased to two-fold from the previous measurement. However, this measurement has shown the gas sensor has repeatability properties without large drift, based on its similar behavior when exposed to hydrogen. In terms of stability properties, the gas sensor can be considered to have good stability, since the sensitivity reduced to 61.16% after six months. Sensitivity decreases with time have also been reported in [55,56].

## 4. Conclusions

A TiO_2-_B_2_O_3_ gas sensor that calcined at 500 °C has shown good performance to low concentrations of hydrogen, as low as 100 ppm at different operating temperatures. The gas sensor also showed an ability to perform at low operating temperatures, to as low as 100 °C. Responses showed that the TiO_2_-B_2_O_3_ gas sensor behaved as a p-type gas sensor, based on decreased currents when exposed to hydrogen. Results showed that highest sensitivity was achieved at an operating temperature of 300 °C with sensitivity values at 1.44, 4.60, and 8.90 for 100 ppm, 500 ppm, and 1000 ppm respectively.
